# Supplementary material for: Substrate-induced conformational dynamics of the dopamine transporter
Source: Nat Commun. 2019 Jun 20;10:2714. doi: 10.1038/s41467-019-10449-w (PMC6586795; doi:10.1038/s41467-019-10449-w)
Supplement: Supplementary file 1 — Supplementary Information [file 41467_2019_10449_MOESM1_ESM.pdf]

SUPPLEMENTARY INFORMATION for:

Substrate-induced conformational dynamics of the dopamine transporter

Nielsen et al.

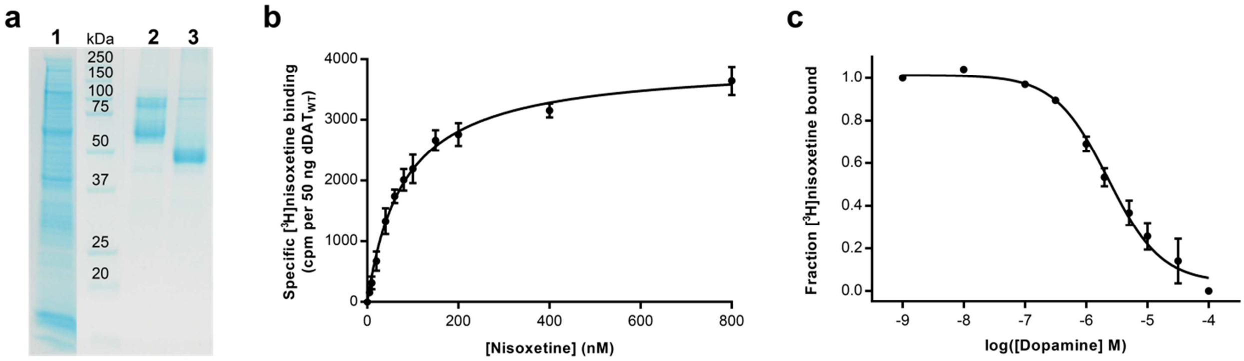

Supplementary Figure 1. Purification and functional characterization of dDAT. a Representative SDS-PAGE showing purity of dDAT eluted from nickel immobilized-metal affinity chromatography. Lane 1, solubilized material before incubation with Ni-NTA resin. Lane 2 and 3 show the purity of dDAT eluted from Ni-NTA resin. The two major bands in lane 2 are caused by heterogeneous glycosylation of dDAT as evident from lane 3, where dDAT appears as one single band after the same sample has been deglycosylated with PNGaseF. However, for the following binding and HDX-MS experiments, dDAT was left glycosylated to retain as wild-type like functionality as possible. **b** Saturation binding of $[^3H]$nisoxetine to purified dDAT with a dissociation constant (K_d) of 80 ± 6 nM. Error bars, s.e.m. of triplicates (*n* = 3). **c** Competitive inhibition of $[^3H]$nisoxetine binding to purified dDAT by unlabeled DA with an inhibition constant (K_i) of 1.7 ± 0.2 µM. Error bars, s.e.m. of triplicates (*n* = 3). Source data are provided as a Source Data file.

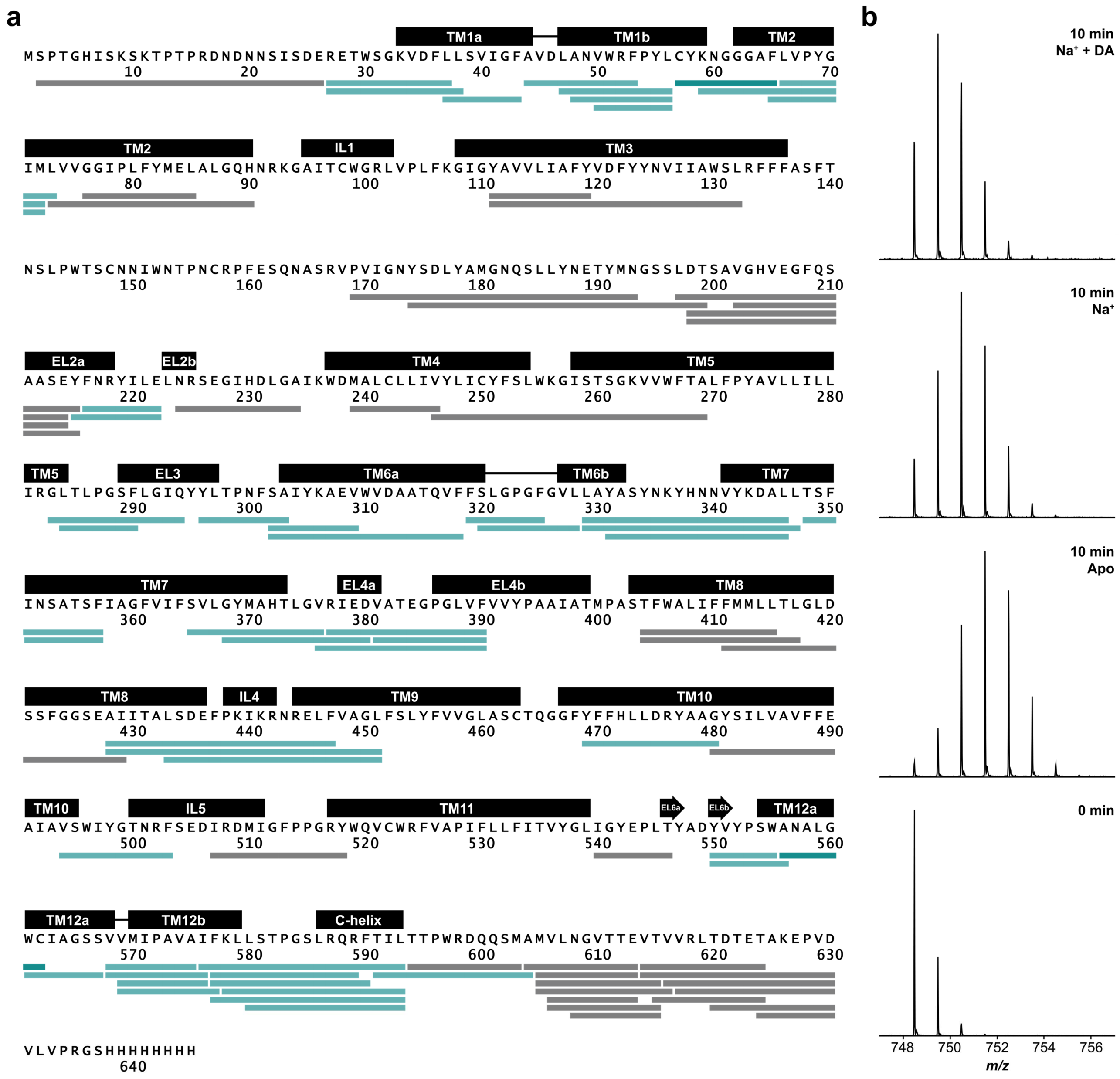

Supplementary Figure 2. Sequence coverage of dDAT. **a** 85 peptides covering 75.5% of the protein sequence (77.2% of the dDAT sequence) were identified following digestion with immobilized pepsin. Individual structural motifs in dDAT are indicated above the protein sequence. Peptides showing a divergence in deuterium uptake of at least one of the sampled states are colored cyan. Peptides, where no signal for the maximum-labeled control was detected, are colored dark cyan (peptide 57 – 65 and peptide 556 - 562). **b** Representative mass spectra for peptide 37-43 from dDAT for undeuterated (0 min) and after 10 min of deuterium exchange. The isotopic envelope shifts to different m/z values as a function of added Na^+ and DA due to the respective degree of deuterium incorporation.

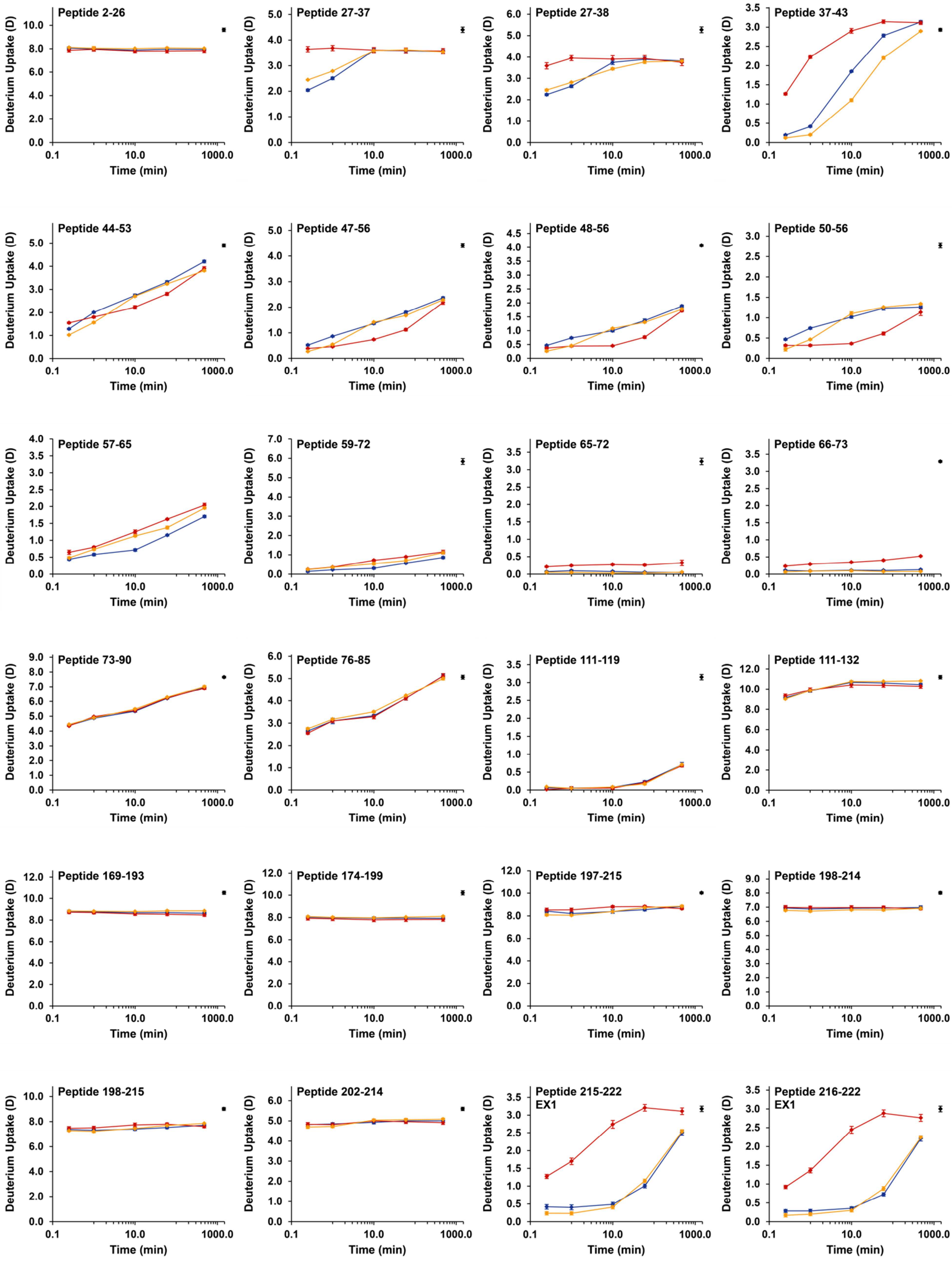
Peptide 2-26
Peptide 27-37
Peptide 27-38
Peptide 37-43
Peptide 44-53
Peptide 47-56
Peptide 48-56
Peptide 50-56
Peptide 57-65
Peptide 59-72
Peptide 65-72
Peptide 66-73
Peptide 73-90
Peptide 76-85
Peptide 111-119
Peptide 111-132
Peptide 169-193
Peptide 174-199
Peptide 197-215
Peptide 198-214
Peptide 198-215
Peptide 202-214
Peptide 215-222 EX1
Peptide 216-222 EX1
Deuterium Uptake (D)
Time (min)

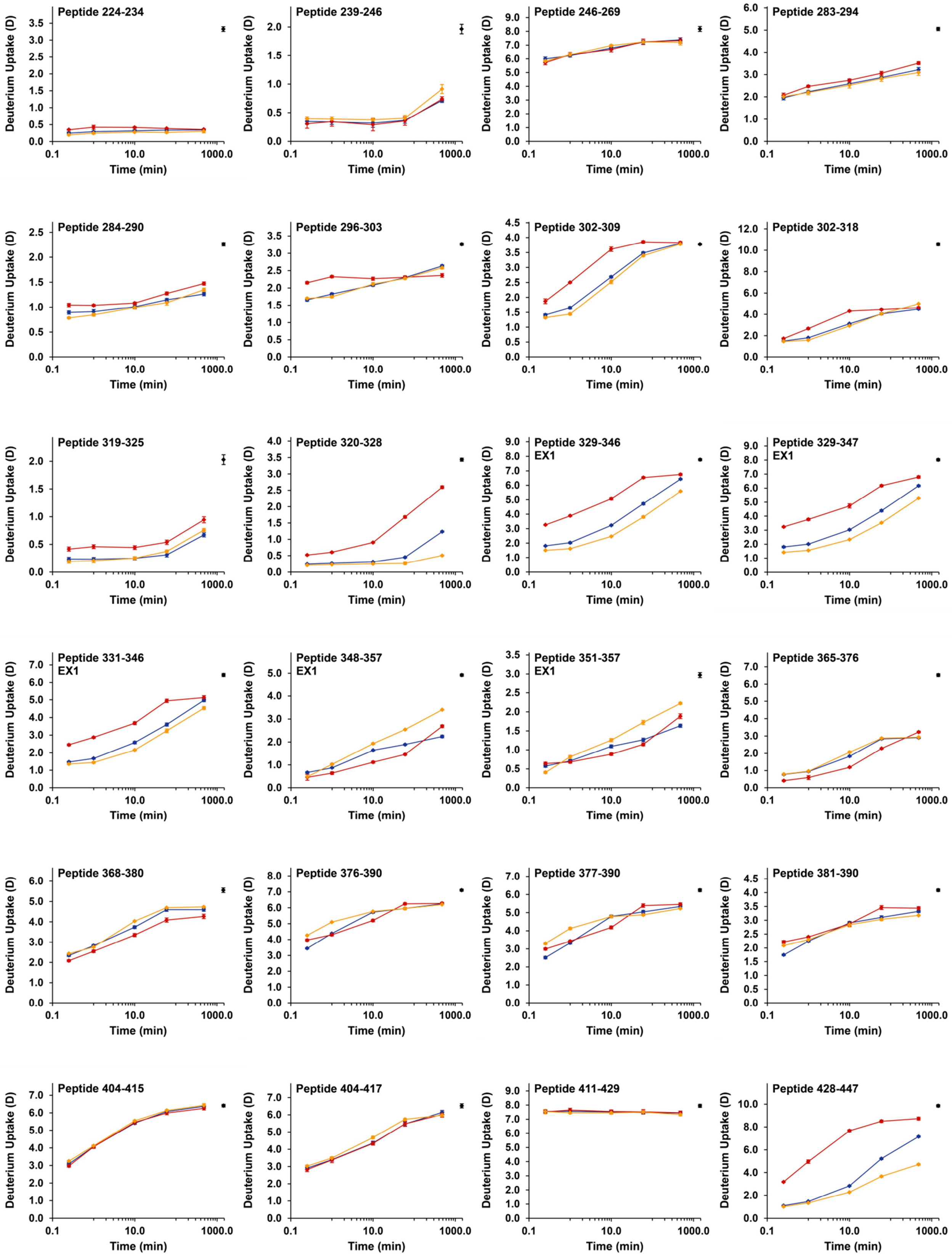
Peptide 224-234
Peptide 239-246
Peptide 246-269
Peptide 283-294
Peptide 284-290
Peptide 296-303
Peptide 302-309
Peptide 302-318
Peptide 319-325
Peptide 320-328
Peptide 329-346 EX1
Peptide 329-347 EX1
Peptide 331-346 EX1
Peptide 348-357 EX1
Peptide 351-357 EX1
Peptide 365-376
Peptide 368-380
Peptide 376-390
Peptide 377-390
Peptide 381-390
Peptide 404-415
Peptide 404-417
Peptide 411-429
Peptide 428-447
Deuterium Uptake (D)
Time (min)

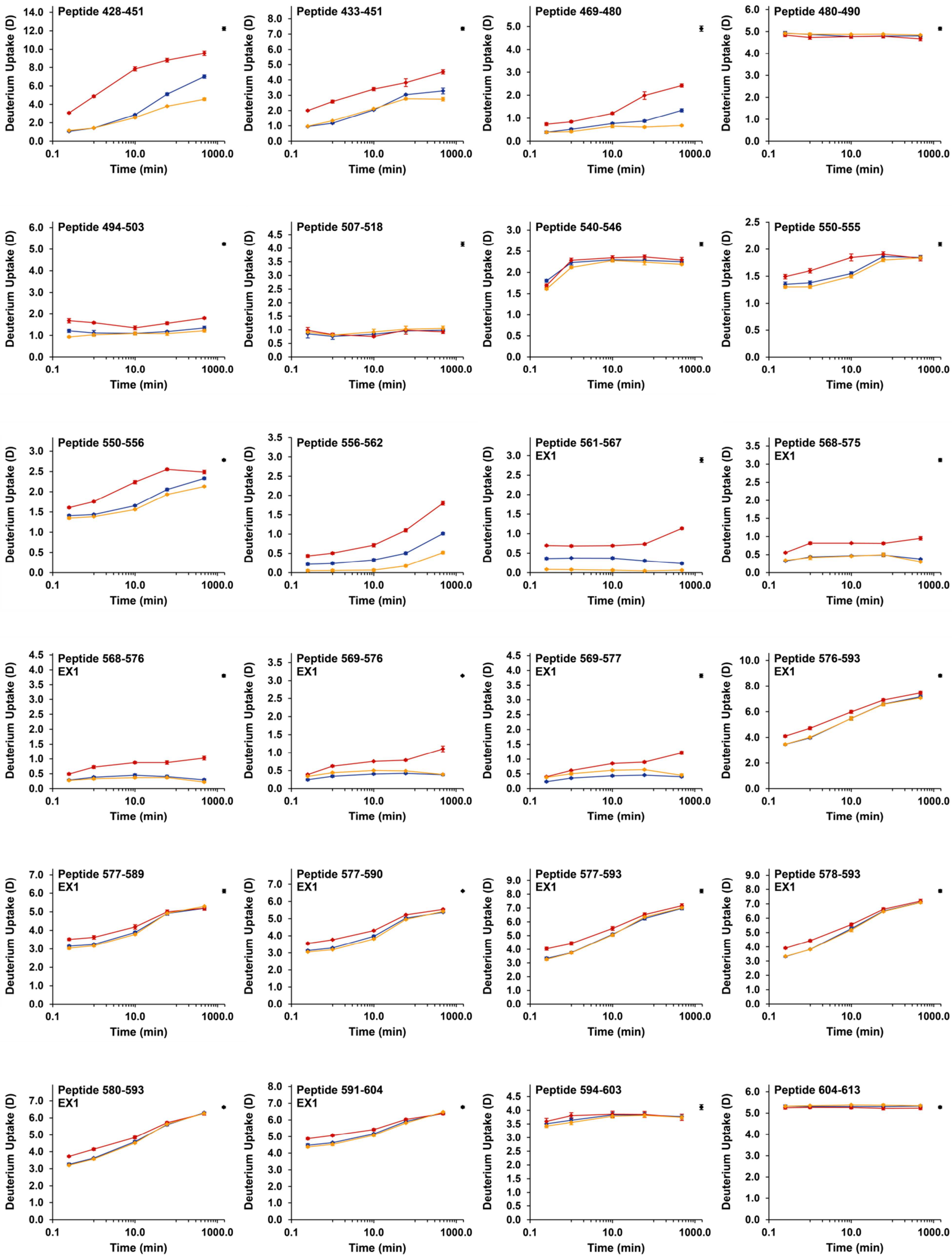
Peptide 428-451
Peptide 433-451
Peptide 469-480
Peptide 480-490
Peptide 494-503
Peptide 507-518
Peptide 540-546
Peptide 550-555
Peptide 550-556
Peptide 556-562
Peptide 561-567
EX1
Peptide 568-575
EX1
Peptide 568-576
EX1
Peptide 569-576
EX1
Peptide 569-577
EX1
Peptide 576-593
EX1
Peptide 577-589
EX1
Peptide 577-590
EX1
Peptide 577-593
EX1
Peptide 578-593
EX1
Peptide 580-593
EX1
Peptide 591-604
EX1
Peptide 594-603
Peptide 604-613
Deuterium Uptake (D)
Time (min)
0.1
10.0
1000.0

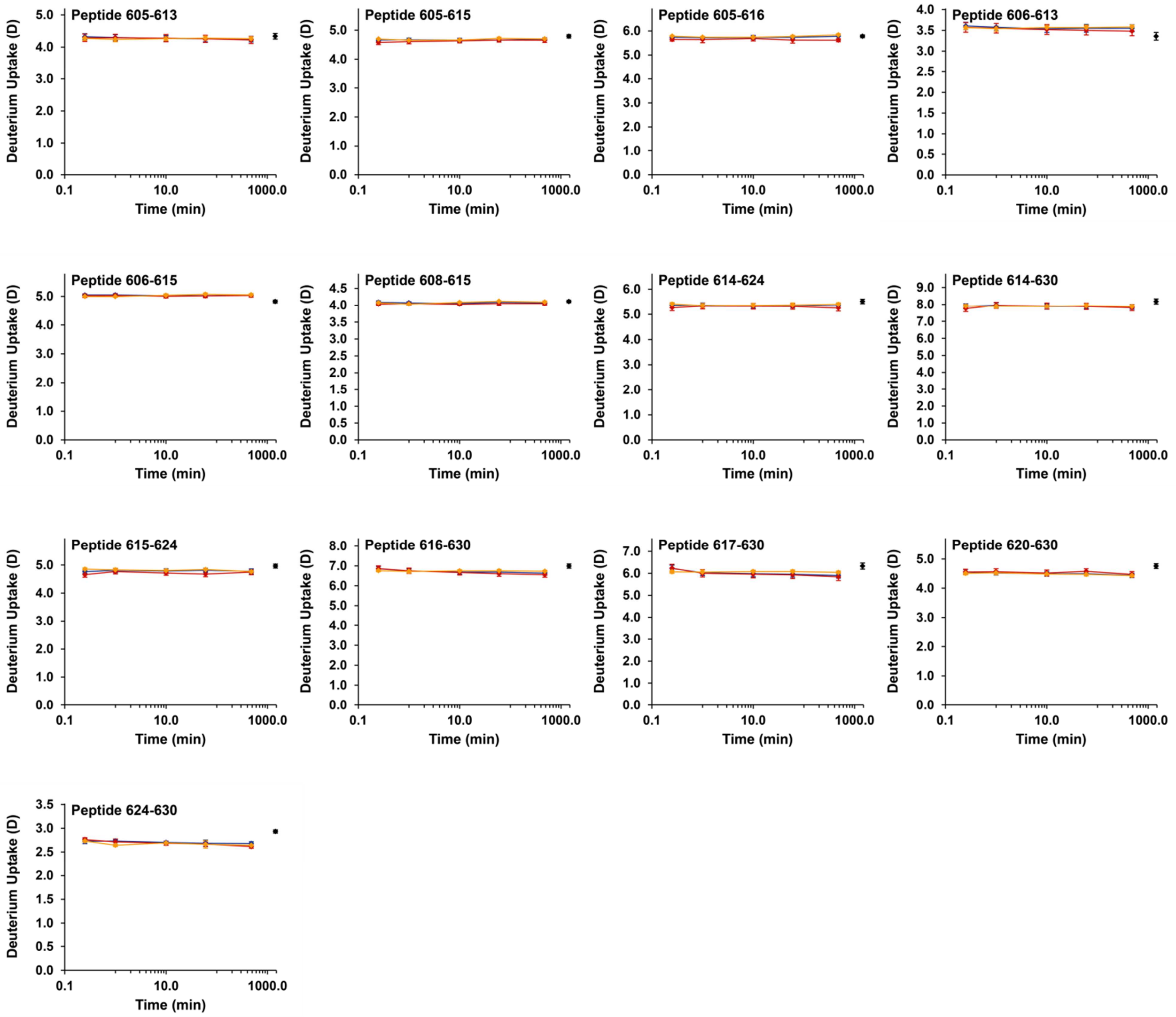

Supplementary Figure 3. Deuterium uptake plots for dDAT in three functional states. The deuterium uptake is plotted as a function of labeling time (*i.e.* 0.25 – 480 min) for all 85 identified dDAT peptides. Red, blue and orange curves represent the apo state, Na^+-bound state, and Na^+- and DA-bound state, respectively. Maximum-labeled control samples are shown as black circles at 1440 min. (*n* = 3 for all time points for the apo state and the Na^+- and DA-bound state, *n* = 6 for all time points for the Na^+-bound state, and *n* = 10 for the maximum-labeled control). Standard deviations are plotted as error bars but are in most instances too small to be visible. Peptides that showed correlated exchange kinetics are marked 'EX1'. Source data are provided as a Source Data file.

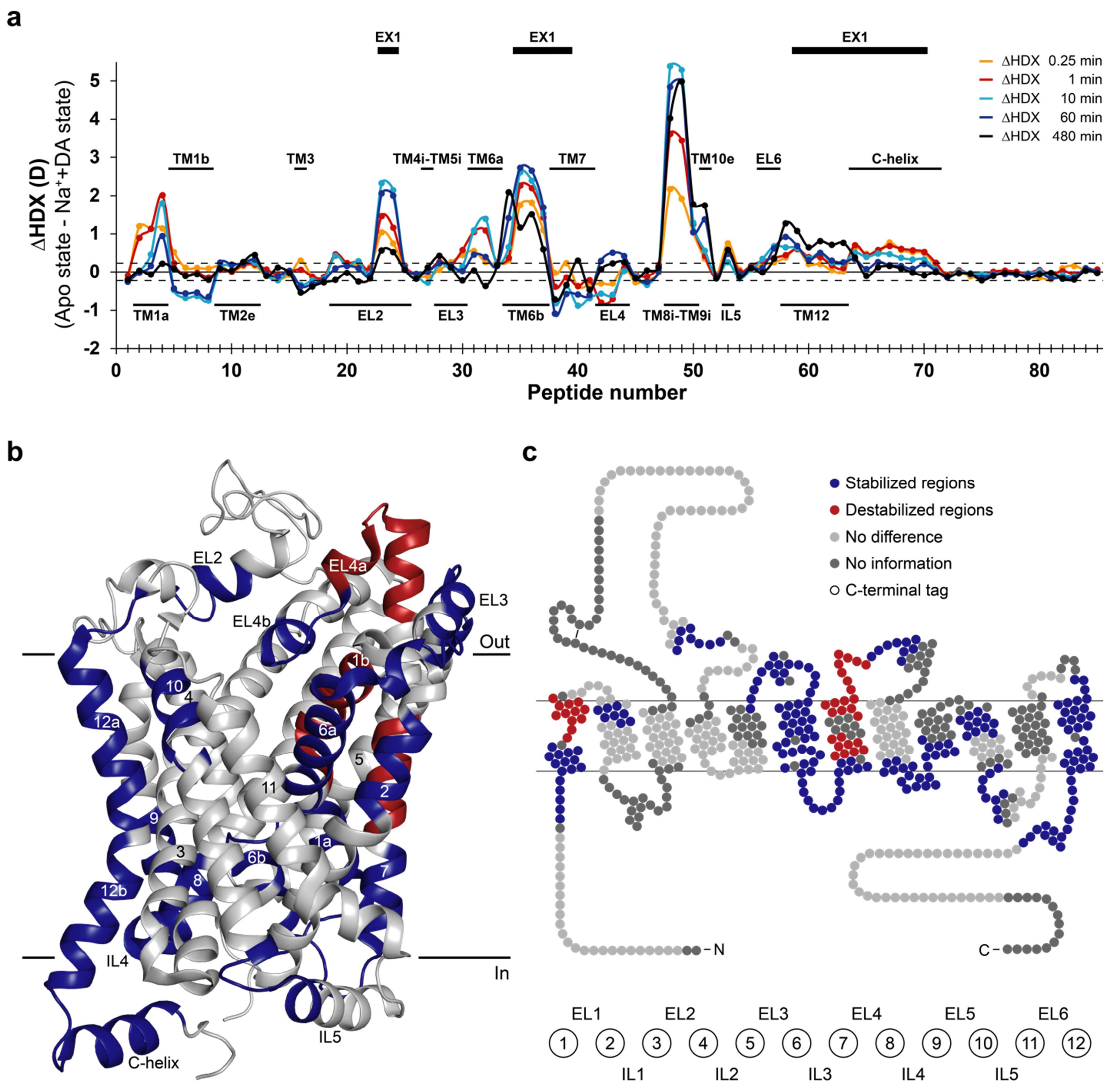

Supplementary Figure 4. Effect of Na^+ and dopamine on the HDX of dDAT compared to the apo state. a Chart showing differences in the average deuterium uptake (ΔHDX) between the apo state and the Na^+- and DA-bound state for the 85 identified peptides at the five sampled time points (orange - 0.25 min; red - 1 min; cyan - 10 min; blue - 1 hour; black - 8 hours). The individual dDAT peptides are arranged along the x-axis starting from the N-terminal and ending on the C-terminal. The peptide number refers to Supplementary Table 2. Positive and negative values indicate reduced and increased HDX, respectively, upon binding of Na^+ and DA. Values represent means of three independent measurements. Structural motifs in dDAT are marked along the x-axis together with regions showing correlated

exchange kinetics (EX1) in at least one of the two states. The dotted lines (±0.23 D) mark a threshold value for significant differences in HDX corresponding to the 95% confidence interval, calculated from the pooled standard deviations for all time points. **b, c** Regions showing significant differences (Student's *t*-test *p*-value < 0.01) in deuterium uptake between the apo state and the Na^+- and DA-bound state for at least two consecutive time points are mapped onto the crystal structure (**b**) (PDB ID: 4XP1) and snake diagram (**c**) of dDAT. Regions are colored red and blue to indicate dDAT segments becoming destabilized (increased HDX) or stabilized (decreased HDX), respectively, upon binding of ions and DA. Regions colored light grey displayed unchanged HDX while regions in dark grey were uncovered by peptide sequences. Wild-type dDAT regions including part of EL2 (residue 162-202) and the N- and C-termini (residue 1-24 and 601-645, respectively) are only marked on the snake diagram in (**c**) as they were not resolved in the crystal structure (**b**) or were truncated in the construct used for crystallization. Source data are provided as a Source Data file.

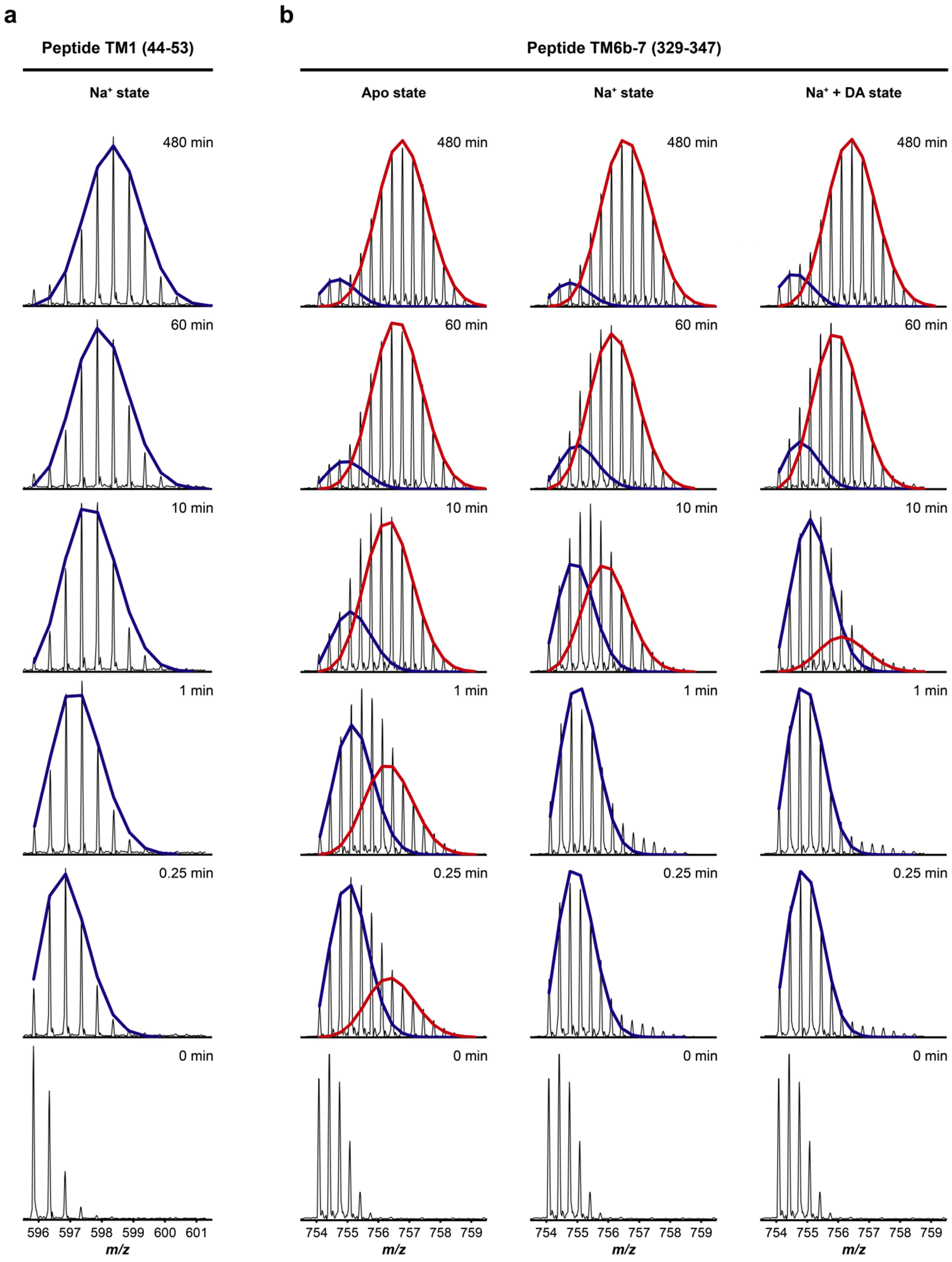

Supplementary Figure 5. Exchange kinetics in dDAT. a Representative mass spectra for peptide 44-53 in the Na^+-bound state at the five sampled time points showing a typical isotopic pattern seen for regions that exchange through the EX2 kinetic regime due to fast local fluctuations that lead to gradual deuteration. One binomial distribution produced the best fit to the spectra (blue). **b** Representative mass spectra for peptide 329-347, which covers TM6b-TM7, are shown for the three states at all sampled time points. The isotopic pattern reveals EX1/EXX exchange

kinetics due to slow cooperative fluctuations that lead to exchange of all backbone amide hydrogens participating in the motion. Two binomial isotopic envelopes gave the best fit to the spectra – yielding a low- (blue) and high-mass (red) population. The rate of translation from the low-mass population to the high-mass population was reduced upon binding of Na^+ relative to the apo state – and even more so by binding of Na^+ and DA combined.

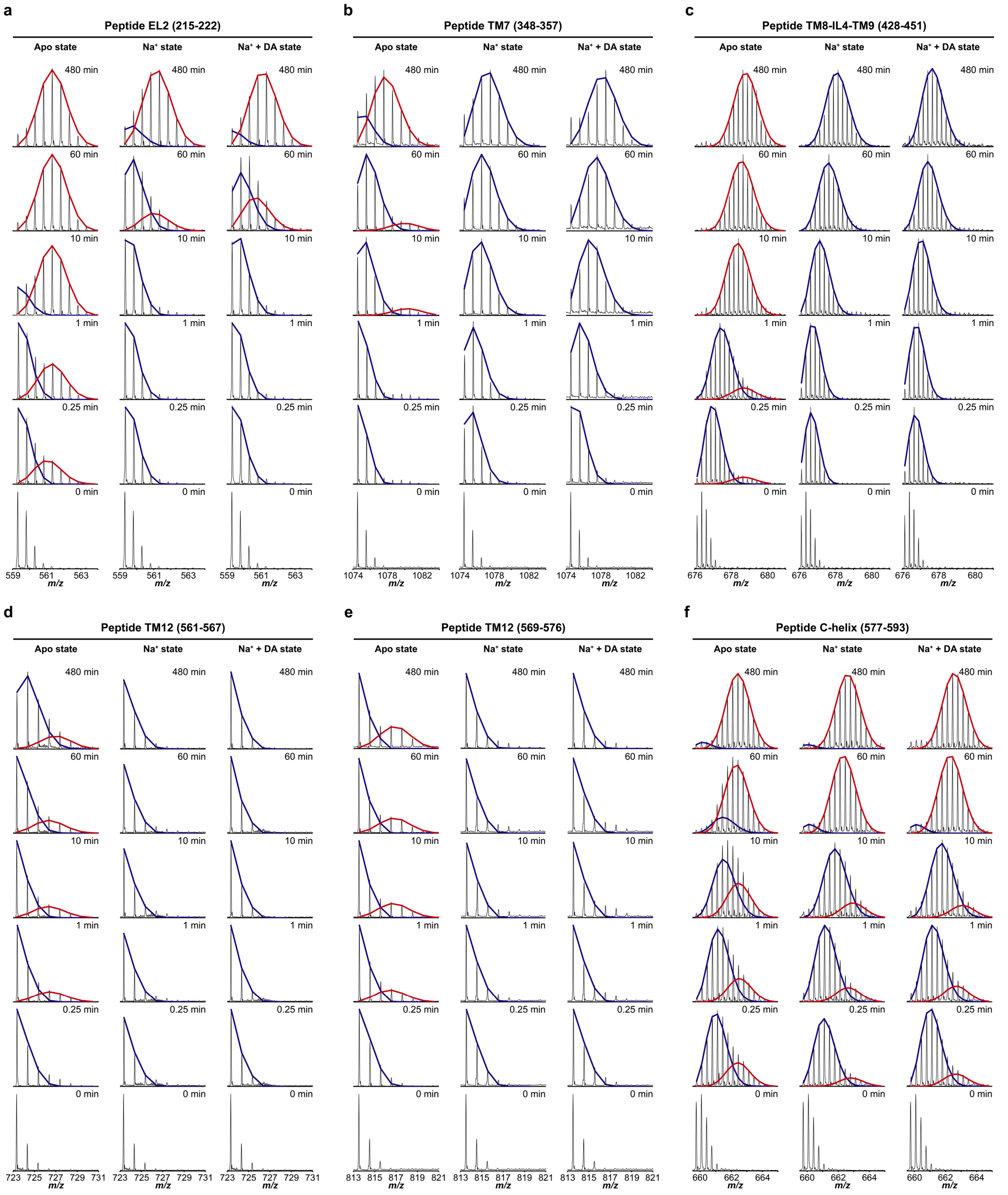

Supplementary Figure 6. EX1/EXX kinetics of regions in dDAT. Representative mass spectra for the three states at all sampled time points for peptides 215-222 (**a**), 348-357 (**b**), 428-451 (**c**), 561-567 (**d**), 569-576 (**e**), and 577-593 (**f**),

which cover EL2, the middle part of TM7, TM8-IL4-TM9, TM12a, TM12b, and the C-helix, respectively. Two binomial isotopic envelopes produced the best fit to the spectra – a low- (blue) and high-mass (red) population.

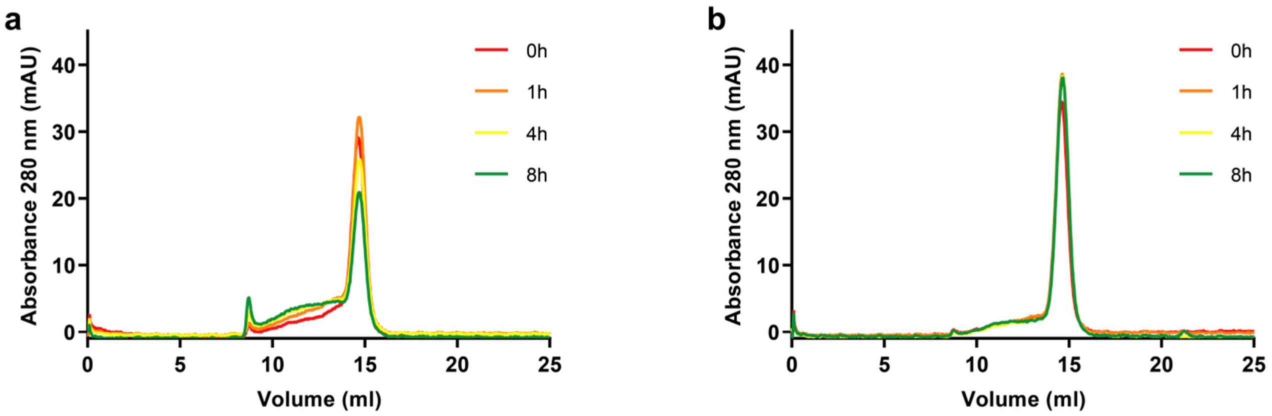

Supplementary Figure 7. Stability of dDAT under sampled conditions. a, b Size-exclusion chromatography (SEC) profiles of purified dDAT incubated at 25°C for the indicated time intervals in buffer A containing 200 mM CsCl (**a**) or 200 mM NaCl (**b**). Source data are provided as a Source Data file.

Supplementary Table 1. HDX data summary table (see Supplementary Dataset 1 for full-scale excel version).

Data Set	Na^+	Apo (Cs^+)	Na^+ + DA
HDX reaction details	40 mM Tris, 5% glycerol, 1 mM DDM, 0.2 mM CHS, 14 µM lipids (POPC:POPE:POPG; weight ratio 3:1:1), 200 mM NaCl. Percent deuterium: 75.2%, pD_{read} = 8.036, 25°C.	40 mM Tris, 5% glycerol, 1 mM DDM, 0.2 mM CHS, 14 µM lipids (POPC:POPE:POPG; weight ratio 3:1:1), 200 mM CsCl. Percent deuterium: 75.2%, pD_{read} = 8.045, 25°C.	40 mM Tris, 5% glycerol, 1 mM DDM, 0.2 mM CHS, 14 µM lipids (POPC:POPE:POPG; weight ratio 3:1:1), 200 mM NaCl, 40 µM DA. Percent deuterium: 75.2%, pD_{read} = 8.036, 25°C.
HDX time course	0.25 min, 1 min, 10 min, 60 min, 480 min	0.25 min, 1 min, 10 min, 60 min, 480 min	0.25 min, 1 min, 10 min, 60 min, 480 min
HDX control samples	Maximum-labeled control using predigested dDAT and the same HDX reaction details as for the Na^+ state.		
Back-exchange (mean / IQR)	32.1% / 9.55%		
# of Peptides	85	85	85
Sequence coverage	75.5% (77.2% of the dDAT sequence)	75.5% (77.2% of the dDAT sequence)	75.5% (77.2% of the dDAT sequence)
Average peptide length / Redundancy	12.44 / 2.17	12.44 / 2.17	12.44 / 2.17
Replicates (biological or technical)	6 (technical)	3 (technical)	3 (technical)
Repeatability	0.0642 (average standard deviation)	0.0807 (average standard deviation)	0.0467 (average standard deviation)
Significant differences in HDX (delta HDX > X D)	Reference state	95% CI: 0.26 D	95% CI: 0.20 D (0.23 D relative to Apo state)

Supplementary Table 2. HDX data table (see Supplementary Dataset 1 for full-scale excel version).

						Uptake - Na^{+} (D)					Uptake - Apo (D)					Uptake - Na^{+} + DA (D)					Uptake - Control Sample (D)	Uptake error (SD) - Na^{+} (D)					Uptake error (SD) - Apo (D)					Uptake error (SD) - Na^{+} + DA (D)					Uptake error (SD) - Control Sample (D)
Peptide #	Start	End	Sequence	Peptide mass (Da)	Retention time (min)	0.25 min	1 min	10 min	60 min	480 min	0.25 min	1 min	10 min	60 min	480 min	0.25 min	1 min	10 min	60 min	480 min	MAX	0.25 min	1 min	10 min	60 min	480 min	0.25 min	1 min	10 min	60 min	480 min	0.25 min	1 min	10 min	60 min	480 min	MAX
1	2	26	SPTGHISKSKTPTPRDNDNNSISDE	2696.2638	3.81	8.06	7.98	7.89	7.96	7.94	7.88	7.96	7.80	7.81	7.83	8.11	8.04	8.02	8.07	8.03	9.61	0.11	0.11	0.12	0.12	0.11	0.15	0.15	0.14	0.15	0.15	0.02	0.16	0.02	0.07	0.03	0.15
2	27	37	RETWSGKVDFL	1336.6775	9.95	2.04	2.50	3.60	3.59	3.55	3.64	3.69	3.61	3.60	3.58	2.44	2.79	3.59	3.62	3.54	4.40	0.05	0.05	0.07	0.08	0.07	0.10	0.10	0.10	0.10	0.10	0.02	0.02	0.04	0.06	0.02	0.11
3	27	38	RETWSGKVDFLL	1449.7616	11.14	2.24	2.63	3.74	3.89	3.82	3.59	3.95	3.91	3.93	3.75	2.45	2.81	3.44	3.77	3.81	5.27	0.05	0.06	0.10	0.11	0.09	0.15	0.12	0.16	0.15	0.15	0.05	0.03	0.05	0.07	0.05	0.13
4	37	43	LLSVIGF	747.4531	12.91	0.19	0.42	1.85	2.78	3.13	1.26	2.22	2.90	3.14	3.12	0.12	0.20	1.10	2.20	2.90	2.93	0.02	0.02	0.02	0.04	0.04	0.03	0.04	0.06	0.04	0.04	0.01	0.02	0.04	0.04	0.02	0.04
5	44	53	AVDLANVWRF	1189.6244	12.58	1.28	2.01	2.74	3.32	4.21	1.55	1.80	2.23	2.81	3.91	1.02	1.56	2.70	3.24	3.81	4.90	0.03	0.04	0.06	0.06	0.06	0.04	0.04	0.06	0.06	0.08	0.02	0.04	0.02	0.04	0.04	0.07
6	47	56	LANVWRFPYL	1277.6921	13.38	0.52	0.86	1.36	1.80	2.37	0.39	0.46	0.74	1.12	2.19	0.27	0.54	1.41	1.68	2.29	4.41	0.04	0.03	0.04	0.05	0.05	0.02	0.03	0.03	0.05	0.07	0.02	0.03	0.03	0.03	0.03	0.08
7	48	56	ANVWRFPYL	1164.6080	12.92	0.47	0.74	1.00	1.36	1.89	0.37	0.44	0.45	0.76	1.72	0.26	0.46	1.08	1.30	1.77	4.07	0.03	0.03	0.05	0.06	0.04	0.03	0.03	0.03	0.05	0.04	0.03	0.03	0.03	0.03	0.03	0.04
8	50	56	VWRFPYL	979.5280	12.87	0.47	0.74	1.02	1.23	1.26	0.32	0.32	0.36	0.61	1.14	0.22	0.46	1.10	1.26	1.34	2.77	0.02	0.03	0.04	0.04	0.03	0.02	0.02	0.02	0.04	0.08	0.04	0.02	0.04	0.02	0.02	0.06
9	57	65	CYKNGGGAF	915.3909	6.10	0.44	0.58	0.72	1.15	1.70	0.65	0.80	1.25	1.63	2.04	0.48	0.73	1.13	1.38	1.95	ND	0.03	0.04	0.04	0.03	0.04	0.06	0.03	0.06	0.03	0.05	0.03	0.03	0.03	0.04	0.03	ND
10	59	72	KNGGGAFLVPYGIM	1422.7330	11.73	0.14	0.22	0.30	0.57	0.86	0.25	0.36	0.71	0.90	1.15	0.24	0.35	0.54	0.70	1.11	5.84	0.06	0.03	0.05	0.05	0.06	0.03	0.03	0.05	0.03	0.10	0.04	0.04	0.07	0.09	0.04	0.15
11	65	72	FLVPYGIM	938.4935	13.15	0.07	0.10	0.07	0.05	0.05	0.21	0.25	0.27	0.26	0.32	0.05	0.05	0.03	0.03	0.05	3.24	0.02	0.02	0.02	0.02	0.03	0.02	0.02	0.02	0.03	0.08	0.03	0.03	0.03	0.02	0.02	0.09
12	66	73	LVPYGIML	904.5092	12.86	0.11	0.09	0.11	0.11	0.13	0.23	0.29	0.35	0.40	0.53	0.06	0.09	0.10	0.08	0.08	3.29	0.02	0.02	0.02	0.02	0.02	0.02	0.02	0.02	0.02	0.02	0.02	0.02	0.04	0.02	0.02	0.03
13	73	90	LVVGGIPLFYMELALGQH	1956.0543	10.98	4.38	4.86	5.34	6.20	6.95	4.35	4.97	5.38	6.23	6.90	4.45	4.90	5.48	6.28	7.01	7.65	0.04	0.04	0.07	0.05	0.05	0.05	0.05	0.10	0.08	0.08	0.03	0.05	0.06	0.03	0.07	0.06
14	76	85	GGIPLFYMEL	1138.5732	9.88	2.65	3.10	3.33	4.11	5.12	2.55	3.10	3.28	4.12	5.12	2.75	3.17	3.51	4.23	5.00	5.06	0.06	0.12	0.06	0.09	0.07	0.05	0.08	0.09	0.09	0.11	0.05	0.03	0.04	0.03	0.06	0.09
15	111	119	YAVVLIAFY	1057.5848	8.06	0.06	0.05	0.08	0.23	0.71	0.02	0.05	0.05	0.21	0.68	0.09	0.05	0.08	0.17	0.71	3.15	0.03	0.04	0.03	0.04	0.06	0.03	0.05	0.04	0.04	0.04	0.00	0.00	0.02	0.03	0.01	0.08
16	111	132	YAVVLIAFYVDFYYNVIIAWSL	2641.3872	10.34	9.14	9.83	10.69	10.62	10.46	9.32	9.93	10.42	10.39	10.29	9.02	9.86	10.76	10.77	10.83	11.19	0.15	0.15	0.16	0.18	0.14	0.18	0.20	0.23	0.20	0.19	0.06	0.08	0.06	0.02	0.04	0.17
17	169	193	PVIGNYSDLYAMGNQSLLYNETYMN	2869.2939	5.31	8.82	8.75	8.68	8.69	8.63	8.72	8.70	8.56	8.54	8.47	8.84	8.81	8.79	8.86	8.85	10.52	0.09	0.10	0.10	0.09	0.09	0.13	0.13	0.13	0.13	0.13	0.03	0.06	0.03	0.08	0.04	0.14
18	174	199	YSDLYAMGNQSLLYNETYMNGSSLDT	2949.2685	5.46	8.03	7.97	7.93	7.94	7.92	7.92	7.88	7.79	7.82	7.82	8.08	8.01	7.97	8.03	8.10	10.21	0.11	0.12	0.13	0.12	0.12	0.15	0.15	0.16	0.17	0.16	0.04	0.09	0.02	0.04	0.08	0.18
19	197	215	LDTSAVGHVEGFQSAASEY	1966.8908	8.90	8.42	8.21	8.38	8.55	8.82	8.52	8.53	8.80	8.82	8.64	8.09	8.05	8.38	8.73	8.85	10.03	0.15	0.09	0.15	0.11	0.07	0.16	0.15	0.10	0.10	0.08	0.09	0.07	0.02	0.07	0.09	0.09
20	198	214	DTSAVGHVEGFQSAASE	1690.7434	7.30	6.94	6.87	6.93	6.93	6.98	6.99	6.96	6.97	6.97	6.90	6.77	6.72	6.81	6.81	6.92	8.01	0.11	0.09	0.08	0.09	0.12	0.13	0.11	0.13	0.11	0.10	0.06	0.08	0.05	0.07	0.11	0.10
21	198	215	DTSAVGHVEGFQSAASEY	1853.8068	8.54	7.36	7.31	7.40	7.53	7.71	7.46	7.50	7.74	7.79	7.61	7.27	7.22	7.45	7.69	7.86	9.01	0.15	0.10	0.11	0.09	0.09	0.14	0.13	0.15	0.11	0.11	0.05	0.10	0.04	0.06	0.04	0.11
22	202	214	VGHVEGFQSAASE	1316.5997	6.13	4.82	4.85	4.93	5.01	5.00	4.83	4.82	5.00	4.96	4.91	4.70	4.72	5.04	5.06	5.08	5.59	0.09	0.07	0.08	0.08	0.07	0.09	0.09	0.09	0.09	0.09	0.07	0.06	0.05	0.07	0.06	0.08
23	215	222	YFNRYILE	1116.5604	9.69	0.42	0.40	0.49	1.01	2.51	1.27	1.70	2.74	3.21	3.11	0.23	0.23	0.41	1.15	2.54	3.18	0.06	0.07	0.06	0.05	0.07	0.06	0.09	0.11	0.09	0.09	0.05	0.05	0.05	0.05	0.05	0.08
24	216	222	FNRYILE	953.4970	8.75	0.28	0.28	0.35	0.72	2.21	0.92	1.36	2.44	2.88	2.76	0.17	0.20	0.30	0.88	2.24	3.00	0.04	0.04	0.04	0.04	0.07	0.05	0.06	0.10	0.09	0.09	0.04	0.04	0.04	0.05	0.04	0.08
25	224	234	NRSEGIHDLGA	1167.5632	5.52	0.25	0.29	0.32	0.34	0.34	0.35	0.42	0.41	0.39	0.36	0.20	0.25	0.28	0.27	0.30	3.33	0.04	0.02	0.03	0.03	0.02	0.02	0.06	0.02	0.02	0.02	0.02	0.03	0.01	0.02	0.05	0.07
26	239	246	MALCLLIV	874.5020	6.67	0.35	0.34	0.32	0.37	0.71	0.31	0.34	0.29	0.36	0.74	0.39	0.39	0.38	0.40	0.91	1.96	0.05	0.04	0.08	0.05	0.03	0.08	0.08	0.11	0.08	0.04	0.03	0.04	0.03	0.05	0.08	0.08
27	246	269	VYLICYFSLWKGISTSGKVVWFTA	2767.4448	6.47	5.99	6.23	6.79	7.21	7.39	5.71	6.31	6.69	7.24	7.33	5.83	6.30	6.97	7.23	7.20	8.17	0.13	0.12	0.14	0.14	0.14	0.15	0.16	0.18	0.20	0.19	0.05	0.15	0.07	0.06	0.19	0.18
28	283	294	GLTLPGSFLGIQ	1201.6707	8.04	1.95	2.23	2.59	2.87	3.22	2.07	2.47	2.74	3.06	3.51	2.01	2.20	2.52	2.82	3.09	5.05	0.09	0.07	0.06	0.07	0.10	0.09	0.05	0.06	0.08	0.07	0.09	0.10	0.13	0.13	0.12	0.07
29	284	290	LTLPGSF	733.4010	10.70	0.89	0.91	1.01	1.15	1.26	1.04	1.04	1.08	1.28	1.47	0.78	0.85	1.00	1.09	1.35	2.26	0.03	0.04	0.04	0.03	0.03	0.03	0.02	0.02	0.03	0.03	0.02	0.03	0.04	0.05	0.03	0.03
30	296	303	YLTPNFSA	911.4389	9.42	1.66	1.82	2.08	2.30	2.63	2.15	2.33	2.27	2.31	2.36	1.70	1.75	2.11	2.28	2.58	3.26	0.04	0.04	0.03	0.04	0.03	0.03	0.03	0.04	0.04	0.05	0.03	0.03	0.04	0.03	0.04	0.03
31	302	309	SAIYKAEV	879.4702	6.32	1.41	1.65	2.69	3.48	3.81	1.87	2.50	3.61	3.85	3.83	1.32	1.44	2.52	3.40	3.79	3.78	0.04	0.04	0.04	0.04	0.03	0.08	0.03	0.08	0.04	0.05	0.03	0.04	0.06	0.04	0.03	0.03
32	302	318	SAIYKAEVWVDAATQVF	1896.9621	13.18	1.50	1.81	3.12	4.05	4.50	1.72	2.66	4.31	4.45	4.63	1.46	1.58	2.92	4.05	5.00	10.56	0.03	0.05	0.06	0.09	0.07	0.06	0.09	0.06	0.07	0.10	0.04	0.04	0.03	0.04	0.04	0.13
33	319	325	FSLGPGF	723.3592	11.63	0.23	0.23	0.25	0.31	0.67	0.42	0.46	0.44	0.54	0.94	0.19	0.20	0.25	0.37	0.76	2.03	0.03	0.03	0.03	0.04	0.04	0.04	0.03	0.03	0.04	0.05	0.03	0.04	0.02	0.03	0.03	0.09
34	320	328	SLGPGFGVL	845.4647	11.94	0.24	0.27	0.31	0.45	1.24	0.52	0.60	0.90	1.68	2.59	0.21	0.23	0.25	0.26	0.50	3.44	0.01	0.01	0.01	0.02	0.02	0.02	0.02	0.02	0.04	0.04	0.01	0.01	0.02	0.04	0.02	0.05
35	329	346	LAYASYNKYHNNVYKDAL	2146.0483	7.39	1.81	2.03	3.23	4.72	6.43	3.26	3.89	5.07	6.53	6.75	1.50	1.62	2.46	3.81	5.59	7.77	0.03	0.06	0.04	0.08	0.05	0.04	0.05	0.09	0.07	0.08	0.02	0.02	0.03	0.08	0.04	0.07
36	329	347	LAYASYNKYHNNVYKDALL	2259.1324	8.43	1.81	2.01	3.03	4.39	6.17	3.24	3.77	4.73	6.17	6.80	1.42	1.56	2.33	3.52	5.28	8.02	0.06	0.04	0.05	0.08	0.07	0.05	0.08	0.15	0.08	0.09	0.03	0.03	0.02	0.04	0.05	0.09
37	331	346	YASYNKYHNNVYKDAL	1961.9272	6.67	1.47	1.67	2.58	3.60	4.98	2.45	2.87	3.68	4.94	5.13	1.35	1.44	2.15	3.25	4.53	6.42	0.04	0.04	0.07	0.09	0.08	0.06	0.05	0.08	0.09	0.11	0.04	0.05	0.04	0.11	0.09	0.08
38	348	357	TSFINSATSF	1073.5029	10.52	0.67	0.87	1.64	1.89	2.24	0.46	0.64	1.12	1.46	2.69	0.48	1.03	1.93	2.54	3.40	4.92	0.04	0.02	0.03	0.03	0.06	0.13	0.06	0.05	0.04	0.06	0.02	0.04	0.04	0.02	0.02	0.05
39	351	357	INSATSF	738.3548	7.36	0.57	0.71	1.09	1.26	1.64	0.64	0.68	0.89	1.15	1.88	0.40	0.82	1.26	1.72	2.22	2.97	0.03	0.04	0.04	0.04	0.04	0.04	0.04	0.03	0.04	0.06	0.03	0.04	0.04	0.05	0.03	0.07
40	365	376	SVLGYMAHTLGV	1246.6380	13.04	0.77	0.94	1.83	2.84	2.90	0.41	0.59	1.19	2.28	3.23	0.78	0.96	2.06	2.87	2.93	6.52	0.03	0.04	0.04	0.05	0.05	0.04	0.11	0.04	0.04	0.06	0.03	0.03	0.06	0.04	0.04	0.08
41	368	380	GYMAHTLGVRIED	1460.7082	7.96	2.35	2.83	3.74	4.60	4.60	2.08	2.55	3.35	4.09	4.27	2.43	2.76	4.03	4.70	4.73	5.55	0.05	0.06	0.07	0.08	0.08	0.05	0.07	0.08	0.10	0.11	0.02	0.04	0.02	0.03	0.03	0.11
42	376	390	VRIEDVATEGPGLVF	1600.8460	11.15	3.45	4.39	5.73	5.95	6.25	3.97	4.31	5.20	6.25	6.28	4.27	5.11	5.77	5.95	6.20	7.10	0.05	0.04	0.07	0.07	0.05	0.07	0.08	0.08	0.07	0.07	0.02	0.02	0.04	0.08	0.02	0.07
43	377	390	RIEDVATEGPGLVF	1501.7776	10.79	2.52	3.33	4.79	5.05	5.34	3.00	3.43	4.19	5.39	5.46	3.30	4.13	4.79	4.88	5.23	6.24	0.07	0.07	0.07	0.08	0.07	0.06	0.05	0.08	0.10	0.08	0.06	0.07	0.06	0.05	0.03	0.08
44	381	390	VATEGPGLVF	988.5229	11.15	1.75	2.24	2.91	3.10	3.32	2.21	2.40	2.87	3.46	3.44	2.09	2.30	2.84	3.04	3.18	4.08	0.03	0.03	0.05	0.06	0.04	0.05	0.03	0.08	0.07	0.06	0.02	0.01	0.08	0.05	0.02	0.05
45	404	415	TFWALIFFMMLL	1531.7971	9.55	3.09	4.07	5.41	6.08	6.37	2.97	4.06	5.46	5.99	6.26	3.25	4.12	5.55	6.14	6.43	6.41	0.07	0.06	0.07	0.07	0.07	0.07	0.10	0.11	0.10	0.09	0.05	0.03	0.05	0.05	0.10	0.08
46	404	417	TFWALIFFMMLLTL	1745.9289	7.09	2.91	3.39	4.36	5.47	6.14	2.82	3.38	4.34	5.48	6.03	3.02	3.49	4.68	5.74	5.98	6.52	0.11	0.08	0.11	0.11	0.12	0.11	0.15	0.10	0.14	0.16	0.06	0.07	0.08	0.06	0.05	0.12
47	411	429	FMMLLTLGLDSSFGGSEAI	1987.9635	9.39	7.56	7.57	7.52	7.48	7.43	7.53	7.65	7.55	7.53	7.46	7.54	7.46	7.45	7.51	7.33	7.95	0.09	0.10	0.08	0.11	0.11	0.12	0.13	0.11	0.16	0.12	0.15	0.07	0.09	0.07	0.10	0.11
48	428	447	AIITALSDEFPKIKRNRELF	2360.3216	10.26	1.10	1.46	2.83	5.22	7.16	3.18	4.96	7.67	8.50	8.73	1.01	1.34	2.28	3.66	4.71	9.85	0.04	0.04	0.05	0.08	0.08	0.05	0.15	0.09	0.11	0.13	0.04	0.05	0.03	0.07	0.07	0.10
49	428	451	AIITALSDEFPKIKRNRELFVAGL	2700.5326	10.97	1.06	1.42	2.86	5.11	7.02	3.07	4.87	7.85	8.78	9.55	1.15	1.43	2.56	3.79	4.56	12.23	0.06	0.05	0.10	0.15	0.17	0.08	0.12	0.22	0.21	0.25	0.08	0.06	0.08	0.07	0.14	0.18
50	433	451	LSDEFPKIKRNRELFVAGL	2231.2426	9.31	0.95	1.17	2.04	3.04	3.28	2.00	2.59	3.40	3.82	4.52	0.97	1.33	2.12	2.77	2.75	7.36	0.03	0.05	0.03	0.05	0.19	0.05	0.10	0.10	0.25	0.14	0.02	0.02	0.04	0.06	0.12	0.10
51	469	480	YFFHLLDRYAAG	1471.7248	11.25	0.39	0.51	0.77	0.88	1.34	0.74	0.84	1.21	1.99	2.42	0.39	0.42	0.65	0.61	0.68	4.90	0.05	0.05	0.04	0.05	0.07	0.06	0.05	0.05	0.17	0.07	0.03	0.04	0.07	0.04	0.03	0.11
52	480	490	GYSILVAVFFE	1243.6489	5.28	4.93	4.87	4.77	4.80	4.80	4.84	4.73	4.78	4.79	4.67	4.92	4.88	4.88	4.89	4.84	5.13	0.08	0.06	0.06	0.08	0.06	0.07	0.08	0.08	0.07	0.09	0.05	0.05	0.02	0.03	0.03	0.07
53	494	503	VSWIYGTNRF	1241.6193	10.62	1.21	1.12	1.10	1.18	1.34	1.68	1.59	1.35	1.56	1.80	0.93	1.03	1.09	1.08	1.22	5.23	0.07	0.11	0.06	0.04	0.08	0.10	0.05	0.09	0.07	0.04	0.04	0.07	0.07	0.08	0.07	0.06
54	507	518	IRDMIGFPPGRY	1420.7285	9.06	0.85	0.76	0.84	0.96	0.98	0.98	0.82	0.75	0.99	0.93	0.91	0.80	0.92	1.02	1.05	4.15	0.15	0.11	0.04	0.05	0.03	0.11	0.05	0.02	0.15	0.06	0.03	0.04	0.10	0.11	0.08	0.08
55	540	546	IGYEPLT	791.4065	8.32	1.80	2.23	2.30	2.28	2.25	1.69	2.28	2.34	2.36	2.29	1.61	2.12	2.28	2.24	2.19	2.67	0.03	0.06	0.04	0.04	0.05	0.04	0.05	0.05	0.04	0.06	0.02	0.03	0.03	0.07	0.02	0.04
56	550	555	YVYPSW	813.3697	11.31	1.35	1.38	1.55	1.86	1.84	1.49	1.60	1.84	1.90	1.83	1.30	1.30	1.50	1.80	1.84	2.09	0.04	0.03	0.03	0.05	0.04	0.04	0.04	0.06	0.04	0.05	0.02	0.03	0.03	0.03	0.03	0.03
57	550	556	YVYPSWA	884.4068	10.95	1.41	1.44	1.66	2.06	2.33	1.61	1.76	2.24	2.56	2.49	1.35	1.38	1.56	1.93	2.13	2.78	0.03	0.02	0.02	0.03	0.03	0.02	0.02	0.04	0.03	0.04	0.03	0.01	0.02	0.02	0.02	0.02
58	556	562	ANALGWC	733.3217	11.01	0.22	0.24	0.33	0.50	1.01	0.43	0.51	0.71	1.10	1.80	0.05	0.06	0.07	0.17	0.52	ND	0.03	0.03	0.03	0.03	0.03	0.03	0.03	0.04	0.04	0.05	0.03	0.03	0.03	0.03	0.03	ND
59	561	567	WCIAGSS	722.3057	8.71	0.36	0.37	0.37	0.30	0.23	0.70	0.68	0.69	0.73	1.13	0.08	0.08	0.06	0.04	0.06	2.89	0.03	0.01	0.03	0.03	0.03	0.02	0.03	0.03	0.03	0.03	0.02	0.02	0.02	0.02	0.02	0.06
60	568	575	VVMIPAVA	798.4673	9.29	0.32	0.43	0.47	0.49	0.38	0.55	0.82	0.82	0.81	0.95	0.34	0.42	0.46	0.50	0.30	3.11	0.02	0.03	0.02	0.03	0.02	0.02	0.03	0.02	0.03	0.05	0.01	0.05	0.02	0.06	0.01	0.04
61	568	576	VVMIPAVAI	911.5514	11.40	0.29	0.38	0.45	0.40	0.29	0.49	0.72	0.88	0.88	1.03	0.28	0.33	0.37	0.37	0.22	3.80	0.03	0.03	0.04	0.04	0.04	0.04	0.05	0.04	0.06	0.06	0.03	0.03	0.03	0.04	0.02	0.04
62	569	576	VMIPAVAI	812.4830	10.81	0.25	0.34	0.41	0.42	0.39	0.39	0.62	0.76	0.79	1.10	0.34	0.44	0.50	0.49	0.39	3.14	0.03	0.03	0.02	0.02	0.03	0.02	0.03	0.01	0.03	0.08	0.01	0.02	0.02	0.01	0.01	0.02
63	569	577	VMIPAVAIF	959.5514	13.35	0.24	0.36	0.43	0.46	0.40	0.40	0.61	0.85	0.90	1.21	0.38	0.50	0.62	0.64	0.45	3.82	0.02	0.02	0.03	0.02	0.03	0.02	0.02	0.02	0.03	0.05	0.02	0.04	0.03	0.02	0.05	0.06
64	576	593	IFKLLSTPGSLRQRFTIL	2089.2412	11.00	3.46	3.97	5.48	6.58	7.16	4.10	4.72	5.98	6.90	7.46	3.46	4.01	5.47	6.56	7.07	8.81	0.07	0.08	0.12	0.11	0.11	0.09	0.11	0.11	0.10	0.12	0.07	0.05	0.15	0.12	0.07	0.10
65	577	589	FKLLSTPGSLRQR	1501.8729	7.23	3.15	3.24	3.89	4.91	5.19	3.51	3.62	4.19	5.00	5.19	3.04	3.17	3.79	4.92	5.30	6.11	0.06	0.06	0.07	0.10	0.08	0.06	0.10	0.12	0.10	0.11	0.07	0.05	0.04	0.05	0.02	0.10
66	577	590	FKLLSTPGSLRQRF	1648.9413	8.77	3.13	3.29	3.97	5.03	5.37	3.55	3.77	4.30	5.22	5.54	3.05	3.19	3.82	4.95	5.42	6.60	0.05	0.04	0.08	0.05	0.03	0.05	0.03	0.05	0.04	0.05	0.03	0.02	0.05	0.05	0.06	0.04
67	577	593	FKLLSTPGSLRQRFTIL	1976.1571	10.50	3.33	3.75	5.09	6.25	6.98	4.04	4.42	5.52	6.52	7.17	3.26	3.74	5.05	6.34	7.01	8.23	0.10	0.08	0.09	0.11	0.11	0.11	0.11	0.14	0.12	0.14	0.09	0.07	0.11	0.09	0.07	0.12
68	578	593	KLLSTPGSLRQRFTIL	1829.0887	9.48	3.32	3.82	5.28	6.49	7.11	3.91	4.43	5.56	6.62	7.19	3.33	3.82	5.19	6.46	7.09	7.89	0.05	0.05	0.10	0.09	0.08	0.06	0.08	0.10	0.10	0.13	0.02	0.04	0.13	0.05	0.05	0.10
69	580	593	LSTPGSLRQRFTIL	1587.9097	9.86	3.25	3.62	4.59	5.60	6.30	3.72	4.15	4.84	5.72	6.26	3.21	3.57	4.51	5.63	6.27	6.63	0.08	0.04	0.07	0.06	0.05	0.05	0.07	0.09	0.07	0.09	0.03	0.05	0.05	0.03	0.02	0.05
70	591	604	TILTTPWRDQQSMA	1646.8086	9.40	4.47	4.63	5.16	5.91	6.41	4.87	5.06	5.41	6.03	6.38	4.37	4.53	5.08	5.82	6.47	6.77	0.09	0.04	0.05	0.06	0.05	0.06	0.06	0.08	0.07	0.08	0.05	0.08	0.06	0.04	0.04	0.06
71	594	603	TTPWRDQQSM	1248.5557	6.89	3.51	3.64	3.83	3.83	3.77	3.60	3.81	3.86	3.85	3.74	3.42	3.56	3.79	3.81	3.75	4.11	0.10	0.09	0.09	0.09	0.08	0.11	0.11	0.11	0.11	0.11	0.06	0.08	0.07	0.06	0.06	0.09
72	604	613	AMVLNGVTTE	1033.5114	8.12	5.32	5.31	5.32	5.31	5.34	5.26	5.27	5.26	5.23	5.23	5.32	5.35	5.39	5.38	5.35	5.28	0.06	0.04	0.08	0.04	0.05	0.06	0.06	0.06	0.07	0.07	0.02	0.03	0.03	0.05	0.02	0.05
73	605	613	MVLNGVTTE	962.4743	7.81	4.32	4.30	4.27	4.26	4.25	4.29	4.29	4.28	4.26	4.22	4.27	4.24	4.26	4.28	4.26	4.34	0.09	0.09	0.09	0.09	0.09	0.11	0.11	0.11	0.11	0.11	0.06	0.07	0.06	0.06	0.06	0.09
74	605	615	MVLNGVTTEVT	1162.5904	8.60	4.66	4.67	4.66	4.66	4.69	4.58	4.61	4.63	4.66	4.66	4.70	4.66	4.66	4.72	4.70	4.79	0.05	0.06	0.06	0.05	0.05	0.07	0.07	0.07	0.07	0.08	0.02	0.05	0.03	0.04	0.02	0.06
75	605	616	MVLNGVTTEVTV	1261.6588	9.91	5.74	5.72	5.73	5.73	5.78	5.65	5.64	5.68	5.62	5.62	5.78	5.74	5.74	5.77	5.84	5.78	0.06	0.05	0.09	0.06	0.10	0.07	0.13	0.10	0.13	0.07	0.03	0.03	0.03	0.05	0.05	0.06
76	606	613	VLNGVTTE	831.4338	5.93	3.61	3.58	3.54	3.55	3.55	3.57	3.55	3.52	3.50	3.48	3.57	3.54	3.57	3.57	3.58	3.36	0.09	0.09	0.09	0.09	0.09	0.11	0.11	0.12	0.11	0.11	0.04	0.06	0.04	0.06	0.05	0.09
77	606	615	VLNGVTTEVT	1031.5499	7.08	5.05	5.05	5.03	5.02	5.05	5.02	5.03	5.00	5.02	5.03	5.00	4.99	5.04	5.07	5.05	4.82	0.04	0.05	0.05	0.05	0.04	0.06	0.06	0.06	0.06	0.06	0.01	0.02	0.05	0.05	0.04	0.05
78	608	615	NGVTTEVT	819.3974	5.21	4.09	4.07	4.05	4.09	4.08	4.03	4.05	4.03	4.05	4.05	4.08	4.03	4.09	4.12	4.10	4.11	0.04	0.03	0.04	0.04	0.04	0.04	0.04	0.04	0.05	0.05	0.04	0.00	0.03	0.02	0.03	0.04
79	614	624	VTVVRLTDTET	1232.6612	7.01	5.35	5.36	5.34	5.35	5.33	5.26	5.33	5.32	5.31	5.25	5.41	5.35	5.36	5.37	5.40	5.51	0.10	0.10	0.10	0.10	0.09	0.12	0.11	0.12	0.11	0.12	0.06	0.09	0.07	0.09	0.06	0.09
80	614	630	VTVVRLTDTETAKEPVD	1871.9840	6.69	7.89	7.95	7.87	7.90	7.86	7.75	7.95	7.90	7.89	7.81	7.91	7.89	7.89	7.93	7.88	8.18	0.15	0.15	0.16	0.15	0.14	0.18	0.18	0.18	0.18	0.18	0.09	0.11	0.09	0.10	0.10	0.15
81	615	624	TVVRLTDTET	1133.5928	6.17	4.77	4.81	4.78	4.81	4.77	4.66	4.77	4.72	4.68	4.74	4.85	4.83	4.80	4.84	4.76	4.96	0.07	0.08	0.08	0.08	0.09	0.09	0.08	0.08	0.09	0.09	0.05	0.06	0.05	0.04	0.05	0.07
82	616	630	VVRLTDTETAKEPVD	1671.8679	5.72	6.85	6.75	6.68	6.68	6.63	6.86	6.75	6.67	6.61	6.55	6.76	6.72	6.75	6.75	6.73	6.99	0.11	0.10	0.10	0.11	0.10	0.13	0.13	0.13	0.13	0.12	0.05	0.08	0.05	0.07	0.05	0.11
83	617	630	VRLTDTETAKEPVD	1572.7995	5.28	6.22	6.03	5.98	5.96	5.90	6.23	6.00	5.96	5.93	5.84	6.07	6.05	6.08	6.08	6.04	6.33	0.15	0.13	0.14	0.13	0.12	0.17	0.16	0.16	0.16	0.16	0.07	0.10	0.07	0.07	0.07	0.14
84	620	630	TDTETAKEPVD	1204.5459	3.77	4.55	4.52	4.49	4.50	4.44	4.55	4.56	4.52	4.58	4.47	4.51	4.53	4.48	4.47	4.43	4.76	0.08	0.07	0.08	0.07	0.08	0.09	0.10	0.10	0.09	0.10	0.05	0.06	0.03	0.05	0.04	0.08
85	624	630	TAKEPVD	758.3810	5.72	2.73	2.73	2.70	2.69	2.68	2.76	2.71	2.68	2.67	2.61	2.73	2.64	2.69	2.66	2.64	2.93	0.05	0.03	0.03	0.07	0.04	0.04	0.06	0.04	0.05	0.03	0.03	0.03	0.02	0.08	0.01	0.04
