# Supplementary material for: Substrate-induced conformational dynamics of the dopamine transporter
Source: Nat Commun. 2019 Jun 20;10:2714. doi: 10.1038/s41467-019-10449-w (PMC6586795; doi:10.1038/s41467-019-10449-w)
Supplement: Supplementary file 3 — Reporting Summary [file 41467_2019_10449_MOESM3_ESM.pdf]

nature research

Corresponding author(s): Claus J. Loland

Last updated by author(s): Apr 29, 2019

Reporting Summary

Nature Research wishes to improve the reproducibility of the work that we publish. This form provides structure for consistency and transparency in reporting. For further information on Nature Research policies, see Authors & Referees and the Editorial Policy Checklist.

Statistics

For all statistical analyses, confirm that the following items are present in the figure legend, table legend, main text, or Methods section.

n/a	Confirmed	
☐	☒	The exact sample size (*n*) for each experimental group/condition, given as a discrete number and unit of measurement
☐	☒	A statement on whether measurements were taken from distinct samples or whether the same sample was measured repeatedly
☐	☒	The statistical test(s) used AND whether they are one- or two-sided<br>*Only common tests should be described solely by name; describe more complex techniques in the Methods section.*
☒	☐	A description of all covariates tested
☐	☒	A description of any assumptions or corrections, such as tests of normality and adjustment for multiple comparisons
☐	☒	A full description of the statistical parameters including central tendency (e.g. means) or other basic estimates (e.g. regression coefficient) AND variation (e.g. standard deviation) or associated estimates of uncertainty (e.g. confidence intervals)
☐	☒	For null hypothesis testing, the test statistic (e.g. *F*, *t*, *r*) with confidence intervals, effect sizes, degrees of freedom and *P* value noted<br>*Give P values as exact values whenever suitable.*
☒	☐	For Bayesian analysis, information on the choice of priors and Markov chain Monte Carlo settings
☒	☐	For hierarchical and complex designs, identification of the appropriate level for tests and full reporting of outcomes
☒	☐	Estimates of effect sizes (e.g. Cohen's *d*, Pearson's *r*), indicating how they were calculated

Our web collection on statistics for biologists contains articles on many of the points above.

Software and code

Policy information about availability of computer code

Data collection	MassLynx (v. 4.1, Waters) was used for controlling the Synapt G2-Si mass spectrometer for the acquisition of MS and MS/MS data.<br>Size-exclusion chromatography data was collected using UNICORN software.
Data analysis	The dDAT model was build using MODELLER9.18.<br>MD simulations were performed using Gromacs2018.<br>GelAnalyzer 2010 was used to analyze the SDS-PAGE gel.<br>GraphPad Prism 7 was used for analysis of data from the ligand binding assays.<br>ProteinLynx Global Server (v. 3.0, Waters) was used for processing MS/MS data.<br>DynamX (v. 3.0, Waters) was used for the processing of HDX-MS data.<br>Microsoft Excel 2010, GraphPad Prism 7 and HX-Express 2.0 were used for analysis of HDX-MS data

For manuscripts utilizing custom algorithms or software that are central to the research but not yet described in published literature, software must be made available to editors/reviewers. We strongly encourage code deposition in a community repository (e.g. GitHub). See the Nature Research guidelines for submitting code & software for further information.

Data

Policy information about availability of data

All manuscripts must include a data availability statement. This statement should provide the following information, where applicable:

- Accession codes, unique identifiers, or web links for publicly available datasets
- A list of figures that have associated raw data
- A description of any restrictions on data availability

HDX-MS data is included in Supplementary Tables 1-2 according to community-based recommendation. Processed DynamX files can be delivered upon request from the authors due to large size. Mass spectrometry data files including processed DynamX files and an overview of the HDX-MS data (Supplementary Tables 1-2) have been uploaded to the PRoteomics IDEntifications (PRIDE) database.

Field-specific reporting

Please select the one below that is the best fit for your research. If you are not sure, read the appropriate sections before making your selection.

☒ Life sciences ☐ Behavioural & social sciences ☐ Ecological, evolutionary & environmental sciences

For a reference copy of the document with all sections, see nature.com/documents/nr-reporting-summary-flat.pdf

Life sciences study design

All studies must disclose on these points even when the disclosure is negative.

Sample size	Samples were measured in at least triplicates (n = 3; n = 6 for the sodium state) .
Data exclusions	Peptides were excluded if they were insufficiently fragmented and/or if the mass error was above 10 ppm.
Replication	Experiments were performed in technical replicates (n = 3; n = 6 for the sodium state).
Randomization	Samples were injected into the LC-MS system according to the measuring time point (i.e. each measuring time point was analyzed to completion before starting a new time point). Samples were injected in a random order within the individual time points.
Blinding	Blinding was not relevant for this study. No live subjects were involved and the sample preparations performed in an experimental laboratory.

Reporting for specific materials, systems and methods

We require information from authors about some types of materials, experimental systems and methods used in many studies. Here, indicate whether each material, system or method listed is relevant to your study. If you are not sure if a list item applies to your research, read the appropriate section before selecting a response.

Materials & experimental systems

n/a	Involved in the study	
☒	☐	Antibodies
☐	☒	Eukaryotic cell lines
☒	☐	Palaeontology
☒	☐	Animals and other organisms
☒	☐	Human research participants
☒	☐	Clinical data

Methods

n/a	Involved in the study	
☒	☐	ChIP-seq
☒	☐	Flow cytometry
☒	☐	MRI-based neuroimaging

Eukaryotic cell lines

Policy information about cell lines

Cell line source(s)	Sf9 cells from Expression Systems were used for the production of the recombinant baculovirus. The HEK293 suspension cell line Expi293F from ThermoFisher Scientific was used for expression of dDAT.
Authentication	The cell lines were commercially bought but not authenticated.
Mycoplasma contamination	The cells were tested and were free from mycoplasma contamination.
Commonly misidentified lines (See ICLAC register)	No commonly misidentified cell lines were used.
